# The Influence of Adjuvant Chemotherapy Dose Intensity on Five-Year Outcomes in Resected Colon Cancer: A Single Centre Retrospective Analysis

**DOI:** 10.3390/curroncol28050342

**Published:** 2021-10-09

**Authors:** Suganija Lakkunarajah, Daniel A. Breadner, Hanbo Zhang, Ellen Yamanaka, Andrew Warner, Stephen Welch

**Affiliations:** 1 Department of Medicine, Schulich School of Medicine & Dentistry, Western University, London, ON N6A 5A5, Canada; suganija.lakkunarajah@lhsc.on.ca; 2 Department of Oncology, Schulich School of Medicine & Dentistry, Western University, London, ON N6A 5W9, Canada; stephen.welch@lhsc.on.ca; 3 London Regional Cancer Program, London Health Sciences Centre, Victoria Hospital, London, ON N6A 5W9, Canada; Ellen.yamanaka@lhsc.on.ca (E.Y.); Andrew.warner@lhsc.on.ca (A.W.); 4 Department of Internal Medicine, Section of Hematology and Oncology, CancerCare Manitoba, Winnipeg, MB R3E 0V9, Canada; hzhang2@cancercare.mb.ca

**Keywords:** dose index, relative dose intensity, colon cancer

## Abstract

There is evidence that achieving a dose intensity > 80% in adjuvant colon cancer treatment improves survival. In total, 192 consecutive patients with resected stage III and high-risk stage II colon cancer that received adjuvant chemotherapy were retrospectively analyzed. Patients who received at least 6 weeks of adjuvant therapy were included. The primary objective was to assess the influence of dose index (DI) and relative dose intensity (RDI) on DFS and OS at 3 and 5 years in patients receiving fluorouracil-based doublet therapy with oxaliplatin (FOLFOX) (5-FU and oxaliplatin assessed separately), or capecitabine monotherapy. In the capecitabine group, DFS rates for 3 and 5 years were 66.7% and 57.6%, respectively, while OS rates were 80.3% and 66.7%, respectively. Those who received FOLFOX had DFS rates of 76.9% and 71.2% at 3 and 5 years, respectively. OS rates were 86.4% and 76.7% at 3 and 5 years, respectively. Median RDI was 73.8% for capecitabine and 76.3% and 85.6% for the oxaliplatin and 5-FU components respectively. Based on a multivariate analysis in patients receiving FOLFOX, those with an oxaliplatin DI > 80% had improvements in DFS and OS compared to those with an oxaliplatin DI of ≤80%. Otherwise, there was no significant difference in DFS or OS when comparing patients who achieved an RDI or a DI of above versus below 80% in the patients receiving adjuvant chemotherapy for resected colon cancer.

## 1. Introduction

Colorectal cancer (CRC) remains the second leading cause of cancer death in developed countries, despite the implementation of early detection and screening programs [1,2,3]. Patients with stage III colon cancer (CC) have benefited significantly from clinical trials, which have substantially reduced the risk of recurrence in this vulnerable group. A few notable changes include 5-fluorouracil (5-FU) based adjuvant chemotherapy, then the addition of oxaliplatin chemotherapy, and the resection of an increased number of lymph nodes for a more complete excision with more accurate staging [4,5,6]. There is substantial research both published and ongoing regarding the timing, duration and choice of different chemotherapy regimens in this population. 

In 2018, the International Duration Evaluation of Adjuvant Chemotherapy (IDEA) collaboration reported its findings looking at three versus six months of adjuvant folinic acid + 5-FU + oxaliplatin (FOLFOX) or capecitabine and oxaliplatin (CAPOX) in patients with resected stage III CC from 12 different countries. They reported 3 months was not non-inferior to 6 months of adjuvant treatment, except in patients with non-T4 and non-N2 disease treated with CAPOX. Considering that three months of adjuvant treatment is sufficient in some populations, it warrants a review of the influence of dose index (DI) and relative dose intensity (RDI) of oxaliplatin-based chemotherapy on disease-free survival (DFS) and overall survival (OS). 

Oxaliplatin has improved outcomes when compared to 5-FU/leucovorin alone in both adjuvant stage III CC and in metastatic CRC [4,7]. Oxaliplatin induced sensory neuropathy are common with FOLFOX and CAPOX and can lead to reduced quality of life, dose reduction, delays, or discontinuation of treatment [8]. Oxaliplatin toxicity increases after three months, especially in the elderly, and the degree of its benefits thereafter varies [9,10,11].

This paper examines the impact of DI and RDI on efficacy outcomes of a population-based cohort of patients with resected stage III CC receiving adjuvant chemotherapy. The primary objective was to assess the influence of DI on DFS and OS for capecitabine and separately for the 5-FU and oxaliplatin components of FOLFOX. Additionally, the influence of RDI and rates of dose delays, dose reductions and 5-FU associated cardiac toxicity were examined. 

## 2. Methods

At a Canadian academic cancer centre, all patients who were seen in consultation for resected stage III CC and received adjuvant chemotherapy between 2006 and 2011 were retrospectively analyzed. Patients were excluded if they developed metastasis within two months of surgery, as these patients were felt to have occult metastatic disease. Patients were excluded from analysis if they received neoadjuvant chemotherapy and/or adjuvant radiation, or if they were determined to have rectal cancer. Patients were only included in the analysis if they received at least three cycles of FOLFOX or two cycles of capecitabine, as it is previously reported that patients with stage III CC who receive limited chemotherapy do not have improved outcomes compared to surgery alone [10,12]. 

**Figure 1:**
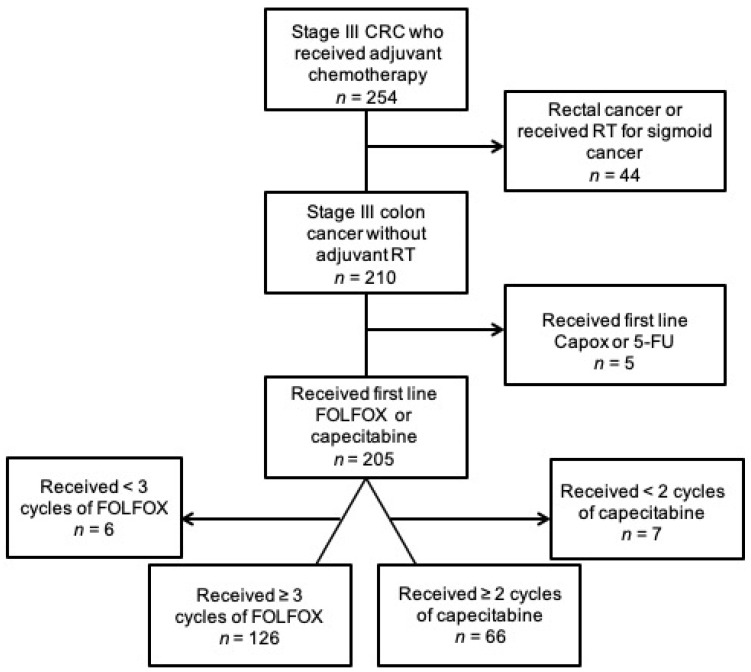
Summary of patient selection.

Analysis of DI and RDI was restricted to patients who received FOLFOX or capecitabine as adjuvant therapy. Patients who switched from FOLFOX to capecitabine had their dose of capecitabine converted to its 5-FU equivalent percentage, and were included in the analysis as patients on FOLFOX. Patients switched from FOLFOX to raltitrexed and oxaliplatin were considered to have stopped therapy and further oxaliplatin was not included in the calculation of oxaliplatin DI and RDI. Patients treated with alternative regimens were not included in analysis, as summarized in Figure 1.

DI and RDI calculations were completed using standard doses for FOLFOX, bolus 5-FU at 400 mg/m^2^, 46 h 5-FU infusion at 2400 mg/m^2^, and oxaliplatin at 85 mg/m^2^. The capecitabine ideal dose was 1250 mg/m^2^ orally twice daily, although there is evidence supporting that 1000 mg/m^2^ orally twice daily is a safer and effective dose in elderly or frail patients, and this dose is commonly used in clinical practice [13,14]. The higher dose was selected as some younger or fit patients elected for capecitabine over intravenous combination chemotherapy where the target dose is 1250 mg/m^2^ orally twice daily.

### Statistical Analysis

DI and RDI were both calculated based on the definitions established by Hryniuk [15,16]. Patients were classified as having a dose reduction if they received less than 90% of the ideal dose during any cycle of their chemotherapy. Dose delays were defined as receiving a cycle of chemotherapy greater than 13 days after the regimen’s scheduled date for either FOLFOX or capecitabine. Descriptive statistics were generated for baseline patient characteristics for all patients and stratified by chemotherapy regimen, compared using the chi-square test, Fisher’s exact test, two-sample t-test or Wilcoxon rank sum test as appropriate. DFS and OS were calculated from the date of surgery to the date of recurrence (DFS only), death due to any cause, or date of last follow-up, whichever occurs first. Kaplan–Meier estimates were generated for DFS and OS, stratified by DI or RDI (≤80% vs. >80%) of capecitabine, 5-FU, and oxaliplatin, and compared using the log-rank test. As a post hoc sensitivity analysis, multivariable Cox proportional hazards regression was performed for DFS and OS adjusted for: chemotherapy regimen (one of: chemotherapy regimen, DI or RDI of capecitabine, 5-FU and oxaliplatin), gender, age, any comorbidity, pathological T stage and N stage. All statistical analyses were performed using SAS version 9.4 software (SAS Institute, Cary, NC, USA) with two-sided statistical testing at the 0.05 significance level.

## 3. Results

### 3.1. Patient Characteristics

Between 2008 and 2012, 254 patients received adjuvant chemotherapy for stage III CRC. Of this group of patients, 192 CC patients received at least a quarter of the planned cycles of FOLFOX (*n* = 126) or capecitabine (*n* = 66) without any adjuvant radiation therapy. Mean ± SD age was 74.5 ± 6.8 years for those who received capecitabine, which was significantly higher than 62.5 ± 8.3 years for those who received FOLFOX (*p* < 0.001). Patients receiving capecitabine also had a significantly higher proportion of having at least one comorbidity compared to FOLFOX (89.4% vs. 77.0%, *p* = 0.036), as shown in Table 1. Dosing changes were more common for patients on FOLFOX compared to capecitabine with 77.0% vs. 60.6% requiring at least 1 dose reduction (*p* = 0.017), 60.3% vs. 45.5% had dose delays (*p* = 0.049), while 63.5% vs. 68.2% completed all planned cycles of therapy (*p* = 0.517). A vast majority of patients initiated therapy with dose intensity of at least 80%, specifically 121 of 126 patients receiving oxaliplatin started treatment above that threshold. Cardiac toxicity (acute coronary syndrome or coronary vasospasm) occurred in 13 patients (6.8%).

The median DI for capecitabine was 72.5% and the median DI for patients receiving FOLFOX was 77.0% and 89.4% for oxaliplatin and 5-FU, respectively. Similarly, the median RDI for capecitabine was 73.8% and the median DI for patients receiving FOLFOX was 76.3% and 85.6% for oxaliplatin and 5-FU, respectively.

DFS and OS data were available at 3-years and 5-years for the vast majority of patients. One patient was lost to follow-up prior to 3 years post-surgery due to moving out of country, and 10 patients were lost to follow-up prior to 5 years due to either not yet reached, or not had medical follow-up visits despite reaching 5 years post-surgery. Median follow-up was 5.8 years for patients receiving capecitabine and 5.7 years for patients receiving FOLFOX.

### 3.2. Disease-Free Survival

**Figure 2:**
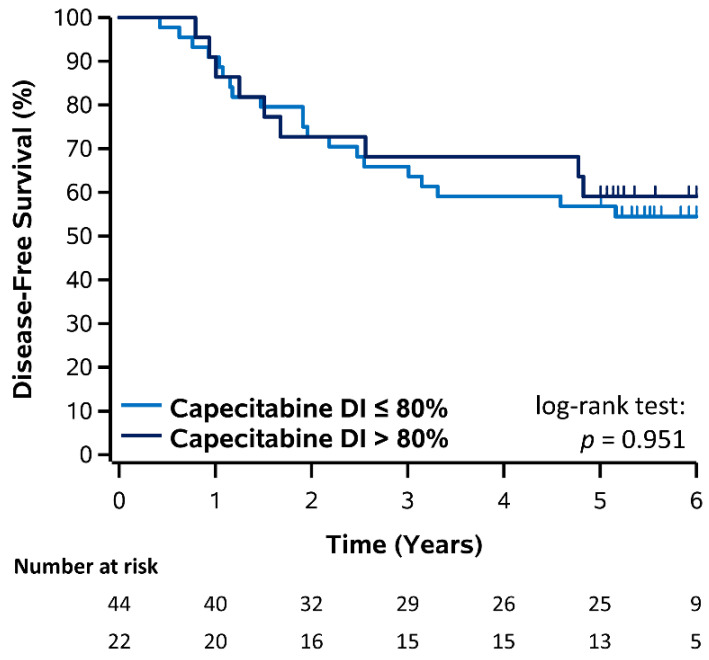
Kaplan–Meier plot of DFS stratified by DI of capecitabine in patients receiving capecitabine.

**Figure 3:**
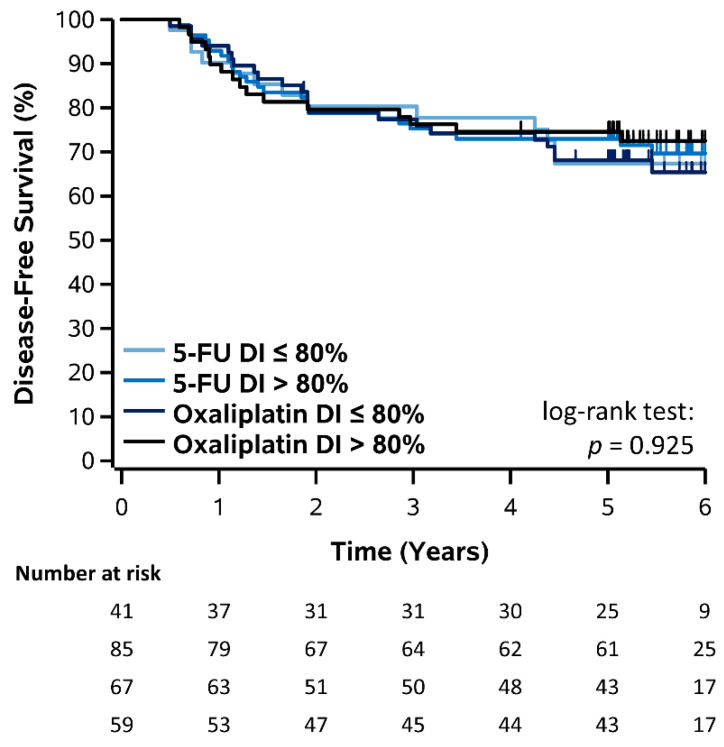
Kaplan–Meier plot of DFS stratified by DI of 5-FU and oxaliplatin in patients receiving FOLFOX.

Kaplan–Meier plots for DFS stratified by DI (≤80% vs. >80%) of capecitabine, 5-FU, and oxaliplatin are shown in Figure 2 and Figure 3 and corresponding estimates summarized in Table 2. For patients who received capecitabine, 3- and 5-year DFS was 65.9% and 56.8% for DI ≤ 80% and 68.2% and 59.1% for DI > 80%, however there was no significant difference (log-rank *p* = 0.951). For patients who received FOLFOX, 3- and 5-year DFS was 80.3% and 67.4% for 5-FU DI ≤ 80% and 75.3% and 72.9% for 5-FU DI > 80%, however this was not significant (log-rank *p* = 0.753). Similarly, 3- and 5-year DFS was 77.4% and 68.1% for oxaliplatin DI ≤ 80% and 76.3% and 74.6% for oxaliplatin DI > 80%, however this was not significant (log-rank *p* = 0.544). Overall, there was also no significant difference comparing all 5-FU and oxaliplatin DI groups and no DFS improvement was observed for patients receiving a DI > 80% (log-rank *p* = 0.925). Results for DFS stratified by RDI (≤80% vs. >80%) of capecitabine, 5-FU, and oxaliplatin were consistent with those for DI and are shown in Supplemental Figures S1 and S2 and Table S1.

Multivariable Cox proportional hazards regression for DFS (adjusting for gender, age, any comorbidity, pathological T stage and N stage) similarly identified no significant differences for patients with capecitabine DI > 80% compared to ≤80% (hazard ratio [HR]: 1.12; 95% confidence interval [CI]: 0.50–2.51; *p* = 0.787) and 5-FU DI > 80% compared to ≤80% (HR: 0.50; 95% CI: 0.24–1.02; *p* = 0.058). However, patients with oxaliplatin DI > 80% had significantly improved DFS compared to ≤80% (HR: 0.36; 95% CI: 0.17–0.75; *p* = 0.006). In contrast, no significant differences were observed for RDI > 80% compared to ≤80% for each of capecitabine, 5-FU and oxaliplatin.

### 3.3. Overall Survival

**Figure 4:**
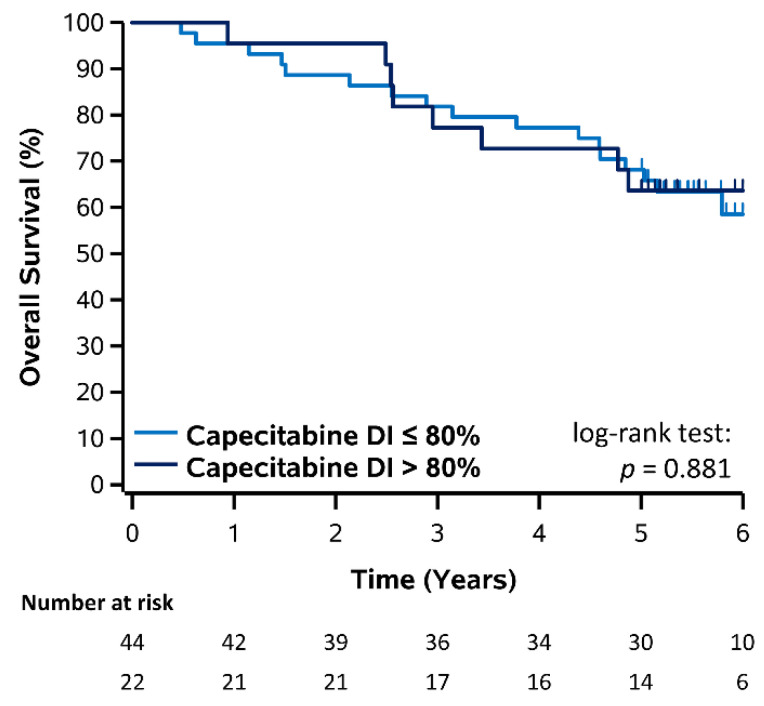
Kaplan–Meier plot of OS stratified by DI of capecitabine in patients receiving capecitabine.

**Figure 5:**
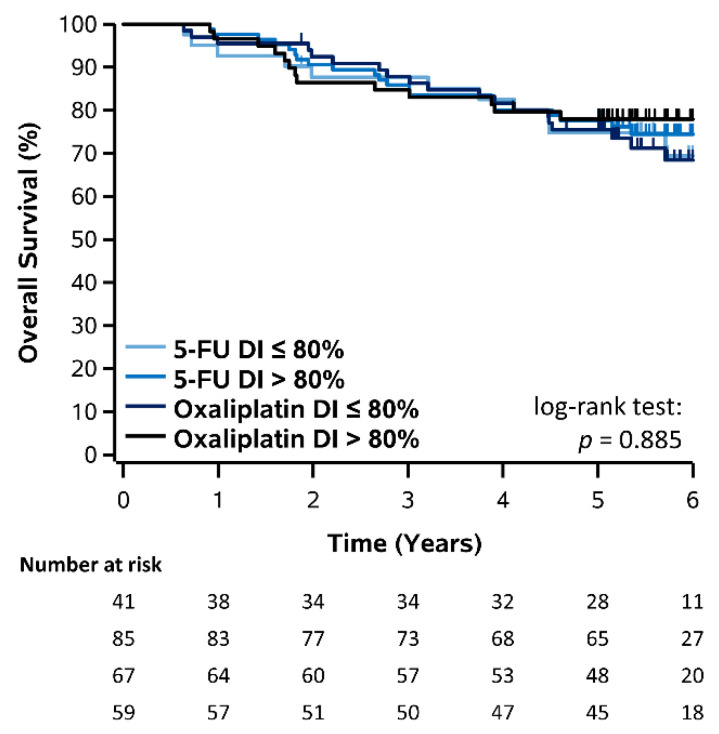
Kaplan–Meier plot of OS stratified by DI of 5-FU and oxaliplatin in patients receiving FOLFOX.

Similarly, Kaplan–Meier plots for OS stratified by DI (≤80% vs. >80%) of capecitabine, 5-FU, and oxaliplatin are shown in Figure 4 and Figure 5 and corresponding estimates also summarized in Table 2. For patients who received capecitabine, 3- and 5-year OS was 81.8% and 68.2% for DI ≤ 80% and 77.3% and 63.6% for DI > 80%, however there was no significant difference (log-rank *p* = 0.881). For patients who received FOLFOX, 3- and 5-year OS was 87.7% and 74.8% for 5-FU DI ≤ 80% and 85.9% and 77.6% for 5-FU DI > 80%, however this was not significant (log-rank *p* = 0.700). Similarly, 3- and 5-year OS was 87.8% and 75.5% for oxaliplatin DI ≤ 80% and 84.7% and 77.9% for oxaliplatin DI > 80%, however this was not significant (log-rank *p* = 0.480). Overall, there was also no significant difference comparing all 5-FU and oxaliplatin DI groups and no OS improvement was observed for patients receiving a DI > 80% (log-rank *p* = 0.885). Results for OS stratified by RDI (≤80% vs. >80%) of capecitabine, 5-FU, and oxaliplatin were consistent with those for DI and are shown in Supplemental Figures S3 and S4 and Table S1.

Multivariable Cox proportional hazards regression for OS also identified no significant differences for patients with capecitabine DI > 80% compared to ≤80% (HR: 1.21; 95% CI: 0.51–2.90; *p* = 0.667) and 5-FU DI > 80% compared to ≤80% (HR: 0.46; 95% CI: 0.21–1.01; *p* = 0.054). Patients with oxaliplatin DI > 80% also had significantly improved OS compared to ≤ 80% (HR: 0.33; 95% CI: 0.14–0.74; *p* = 0.007). In contrast, no significant differences were observed for RDI > 80% compared to ≤80% for each of capecitabine, 5-FU and oxaliplatin.

## 4. Discussion

This single-centre retrospective study reports no significant difference in survival outcomes at 3 or 5 years based on DI or RDI for capecitabine, as monotherapy, or the 5-FU component of FOLFOX in the adjuvant setting for stage III CRC. Oxaliplatin RDI did not significantly affect outcomes. However, there was an improvement in DFS and OS in patients with an oxaliplatin DI above 80% compared to those with a DI of less than 80%, based on multivariable analysis.

Much variability exists in the literature as to how to define and calculate DI (also referred to as dose intensity). The current study selected the definition proposed by Hryniuk, which calculates the actual dose received against the ideal dose based on the regimen [15]. Similarly, there are multiple definitions for calculating RDI [15,17], however the current study selected the definition proposed by Hryniuk. All definitions calculate RDI based on the ideal dose over the target delivery time, however there is a variation on whether early cessation is incorporated, although it always leads to a reduction in DI. The DI definition used in the current study does not take into consideration the time taken to complete the treatment and may not accurately represent those who had dose delays. This investigation emphasized DI over RDI results as there is good evidence that a patient receiving a full dose of FOLFOX or CAPOX for 3 months and then stopping treatment would have a different level of benefit than a patient receiving a 50% dose of FOLFOX or CAPOX for 6 months, and completing on schedule. Both scenarios may translate to an RDI of 50%, depending on the definition used.

RDI has been shown to have an impact on outcomes in a number of cancers, including breast, lung, lymphoma, and CRC [16,18,19,20,21,22]. In the metastatic CRC setting, Nakayama reported an RDI of greater than 80% of irinotecan in folinic acid + 5-FU + irinotecan (FOLFIRI) based chemotherapy was associated with a significant improvement in response rate (RR), disease control rate (DCR), progression-free survival (PFS), and OS; while an RDI of greater than 79% of oxaliplatin in FOLFOX demonstrated a significant improvement in DCR in a study population of only 30 patients [16]. In stage III CC patients, Morris reported failing to complete more than three cycles of adjuvant therapy led to outcomes inferior to surgery alone, while patients completing four or more cycles had superior outcomes [12]. Two additional studies by Aspinall and Ho revealed an RDI of greater than 70% was associated with improved outcomes in stage III CC [23,24]. Aspinall noted a 5-year OS improvement of 15.8% and a 3-year DFS increase of 13.4% when RDI was greater than 70%. However, Ho’s abstract was only reporting on capecitabine monotherapy, and Aspinall’s analysis, although well powered, only included American veterans, so neither study reported outcomes for oxaliplatin based chemotherapy in a general population.

There is now evidence that extending adjuvant chemotherapy beyond 3 months is not beneficial in some circumstances and can lead to increased toxicity [25,26]. In practice, physicians try to balance toxicity with the objective of reducing CC recurrence risk. This is especially true with oxaliplatin chemotherapy, which carries a considerable risk of acute and chronic peripheral neuropathy. This retrospective analysis seeks to inform patients and physicians of the trade-off in CC recurrence risk with toxicity and safety in patients requiring a dose reduction.

The two regimens assessed in this analysis were FOLFOX and capecitabine monotherapy, as CAPOX was not reimbursed during the examined period. 5-FU and oxaliplatin doublet is considered the standard of care for adjuvant therapy in high-risk stage II or stage III CC, in medically fit patients. This report reveals a DFS and OS benefit in patients receiving at least 80% of the intended oxaliplatin in a population of 126 patients receiving FOLFOX adjuvant chemotherapy for CC. There were no other differences in clinical outcomes based on oxaliplatin RDI or 5-FU DI or RDI in this group of patients receiving FOLFOX. Considering the growing evidence that 3 months of adjuvant doublet chemotherapy is equally as effective as 6 months, in certain circumstances, perhaps the more poignant question is to examine the influence of DI or RDI in the first three months of treatment and the time from surgery to initiating adjuvant chemotherapy. A larger population would also allow subgroup analyses in those with certain risk factors, such as pathological T4 or N2 resected disease.

The 66 patients receiving capecitabine monotherapy in this study were significantly older than the FOLFOX population. This was expected as monotherapy is typically reserved for older or frail patients or those with contraindications to oxaliplatin [14]. This study did not include a comparison between doublet and monotherapy, as it is well established that capecitabine monotherapy is inferior to oxaliplatin-based regimens [27]. This study contained a subset of patients receiving capecitabine monotherapy and there is limited data on dose intensity in the adjuvant setting for older or frail patients, and data on monotherapy predating the widespread use of oxaliplatin does not generally apply. Neugut et al. had examined patients ≥ 65 years old receiving 5-FU based chemotherapy and observed that early cessation of treatment was associated with a doubling of mortality rates compared to patients completing 6 months of treatment [10]. In this retrospective observational study we report no significant difference in survival outcomes when a DI of less than 80% is achieved, which is reassuring for patients and clinicians when capecitabine dose reductions and/or early cessation are needed.

There are several limitations in this study; most particularly it is a retrospective single centre analysis based on a smaller population size. Therefore, this study may not have been sufficiently powered to detect the influence of dose intensity on recurrence. A clear strength is that three-year DFS and OS data were available on all but one patient, and a vast majority of patients had five-year data available. Collaboration with other Canadian institutions would allow for a more sufficiently powered examination of the effect of DI on 5-year OS and DFS in patients with resected CC and validate the results of the current study.

## 5. Conclusions

In 126 patients with resected high-risk stage II and stage III CC, receiving adjuvant FOLFOX, patients receiving at least 80% of the ideal dose of oxaliplatin had improvements in DFS and OS, compared to those with a DI of less than 80%. Oxaliplatin RDI did significantly influence DFS or OS. There was no difference in survival outcomes in patients receiving FOLFOX with a 5-FU DI or RDI of at least 80% compared to those with a 5-FU DI or RDI of less than 80%. In 66 patients receiving adjuvant capecitabine monotherapy there were no differences in recurrence or survival outcomes based on DI or RDI of at least 80% compared to those with a 5-FU DI or RDI of less than 80%. Considering recent evidence challenging the need for 6 months of adjuvant chemotherapy for some patients, examining the influence of DI and RDI in the first three months of adjuvant therapy would be more applicable, in certain populations. Pooling data from multiple institutions is needed to better evaluate the influence of DI and RDI on adjuvant CC survival outcomes.

## Figures and Tables

**Table 1:** Baseline patient characteristics (*n* = 192).

Characteristic	All Patients (*n* = 192)	Capecitabine (*n* = 66)	FOLFOX (*n* = 126)	*p*-Value
Gender—*n* (%)				0.482
Male	98 (51.0)	36 (54.6)	62 (49.2)	
Female	94 (49.0)	30 (45.5)	64 (50.8)	
Age—median (IQR)	67.7 (60.5, 73.0)	75.1 (71.7, 78.9)	63.8 (57.5, 69.1)	<0.001
Resection to discharge (days)—median (IQR)	6 (5, 8)	7 (6, 9)	6 (4, 8)	0.004
Post-operative hospital re-admission—*n* (%)	14 (7.3)	8 (12.1)	6 (4.8)	0.080
Surgery to medical oncology referral consult (days)—median (IQR)	21 (14, 30)	23 (15, 31)	21 (13, 28)	0.226
Comorbidity: any—*n* (%)	156 (81.3)	59 (89.4)	97 (77.0)	0.036
Perioperative complications—*n* (%)	49 (25.5)	25 (37.9)	24 (19.1)	0.005
Pathological T stage—*n* (%)				0.803
T0	1 (0.5)	0 (0)	1 (0.8)	
T1	7 (3.7)	2 (3.0)	5 (4.0)	
T2	12 (6.3)	3 (4.6)	9 (7.1)	
T3	93 (48.4)	36 (54.6)	57 (45.2)	
T4	79 (41.2)	25 (37.9)	54 (42.9)	
Pathological N stage—*n* (%)				0.605
N0	30 (15.6)	12 (18.2)	18 (14.3)	
N1	111 (57.8)	39 (59.1)	72 (57.1)	
N2	51 (26.6)	15 (22.7)	36 (28.6)	
Status—*n* (%)				0.083
Alive with disease	5 (2.6)	2 (3.0)	3 (2.4)	
Alive without disease	129 (67.2)	38 (57.6)	91 (72.2)	
Death with disease	43 (22.4)	17 (25.8)	26 (20.6)	
Death without disease	15 (7.8)	9 (13.6)	6 (4.8)	
Death—*n* (%)	58 (30.2)	26 (39.4)	32 (25.4)	0.045
Recurrence—*n* (%)	59 (30.7)	23 (34.9)	36 (28.6)	0.371
Median follow-up (years)—median (95% CI)	5.74 (5.52, 5.96)	5.79 (5.46, 6.08)	5.74 (5.45, 6.01)	0.758

IQR = Interquartile range.

**Table 2:** The 3 and 5-year DFS and OS stratified by DI of capecitabine, 5-FU and oxaliplatin.

	**3-Year DFS (%)**		**5-Year DFS (%)**		**Log-Rank *p*-Value**
Dose Intensity	≤80%	>80%	≤80%	>80%	
Capecitabine	65.9%	68.2%	56.8%	59.1%	0.951
5-FU	80.3%	75.3%	67.4%	72.9%	0.753
Oxaliplatin	77.4%	76.3%	68.1%	74.6%	0.544
	**3-Year OS (%)**		**5-Year OS (%)**		**Log-Rank *p*-Value**
Dose Intensity	≤80%	>80%	≤80%	>80%	
Capecitabine	81.8%	77.3%	68.2%	63.6%	0.881
5-FU	87.7%	85.9%	74.8%	77.6%	0.700
Oxaliplatin	87.8%	84.7%	75.5%	77.9%	0.480

## Data Availability

Data from this study are available to researchers through a data access process.

## Supplementary Materials

The following are available online at https://www.mdpi.com/article/10.3390/curroncol28050342/s1, Table S1: The 3 and 5-year DFS and OS are reported for those who achieved greater than 80% RDI for the respective therapeutic versus those who did not exceed 80% RDI, Figure S1: Kaplan–Meier plot of DFS stratified by RDI of capecitabine in patients receiving capecitabine, Figure S2: Kaplan–Meier plot of DFS stratified by RDI of 5-FU and oxaliplatin in patients receiving FOLFOX, Figure S3: Kaplan–Meier plot of OS stratified by RDI of capecitabine in patients receiving capecitabine, Figure S4: Kaplan–Meier plot of OS stratified by RDI of 5-FU and oxaliplatin in patients receiving FOLFOX.
