# Supplementary material for: The Influence of Adjuvant Chemotherapy Dose Intensity on Five-Year Outcomes in Resected Colon Cancer: A Single Centre Retrospective Analysis
Source: Curr Oncol. 2021 Oct 9;28(5):4031–41. doi: 10.3390/curroncol28050342 (PMC8535138; doi:10.3390/curroncol28050342)
Supplement: Supplementary file 1 [file curroncol-28-00342-s001.zip › curroncol-1281446-supplementary.pdf]

Table S1. The 3 and 5-year DFS and OS are reported for those who achieved greater than 80% RDI for the respective therapeutic versus those who did not exceed 80% RDI.

		**3-year DFS (%)**		5-year DFS (%)		P value
		≤80%	> 80%	≤80%	>80%	
Capecitabine	RDI	67.4%	65.2%	55.8%	60.9%	0.820
Oxaliplatin	RDI	76.9%	76.8%	72.8%	68.8%	0.868
5-FU	RDI	79.9%	75.2%	77.7%	67.6%	0.512
		3-year OS (%)		5-year OS (%)		P value
		≤80%	> 80%	≤80%	> 80%	
Capecitabine	RDI	79.1%	82.6%	62.8%	73.9%	0.560
Oxaliplatin	RDI	87.7%	84.6%	75.4%	78.6%	0.523
5-FU	RDI	91.1%	83.9%	79.7%	75.0%	0.691

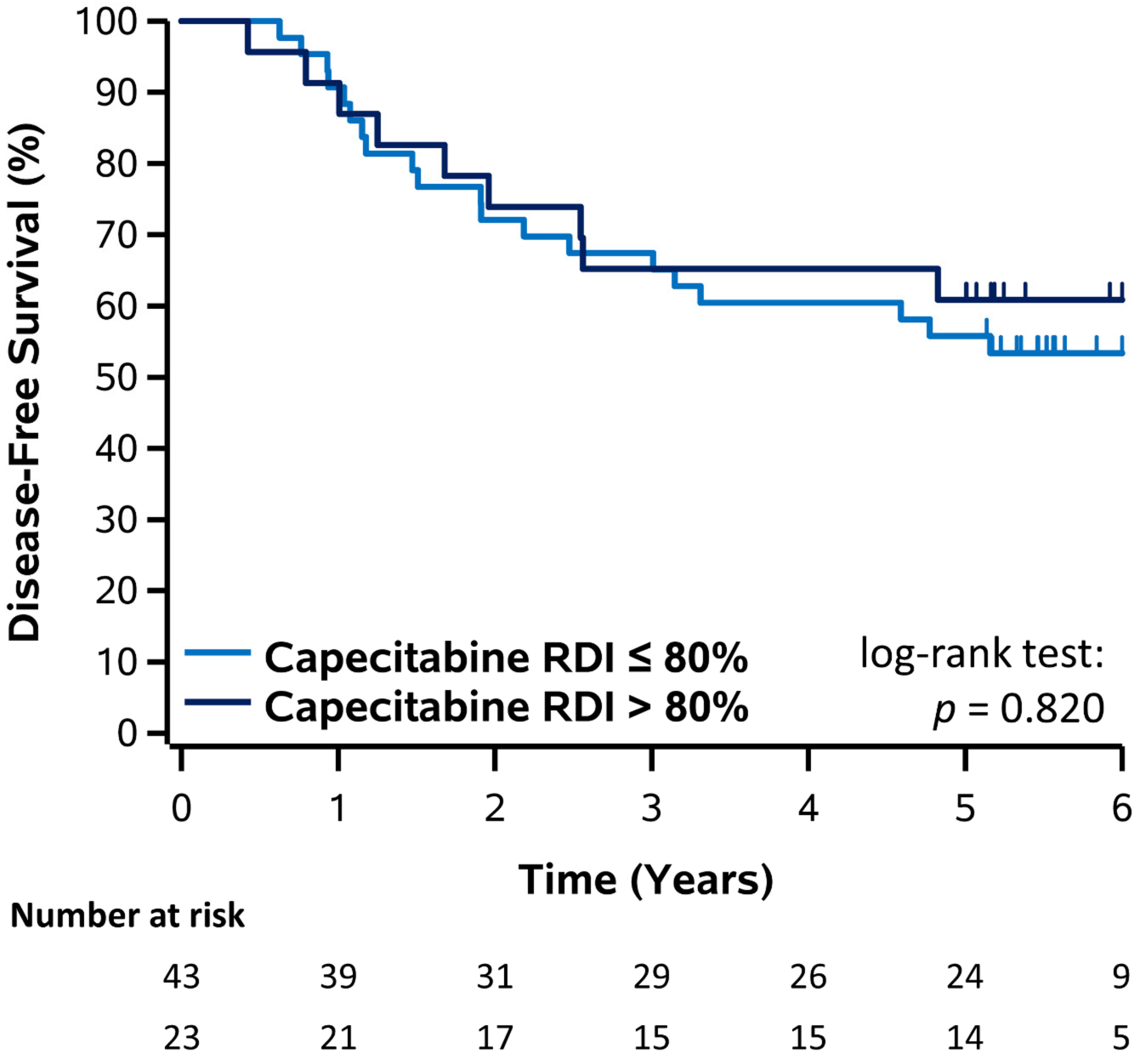

Figure S1. Kaplan–Meier plot of DFS stratified by RDI of capecitabine in patients receiving capecitabine.

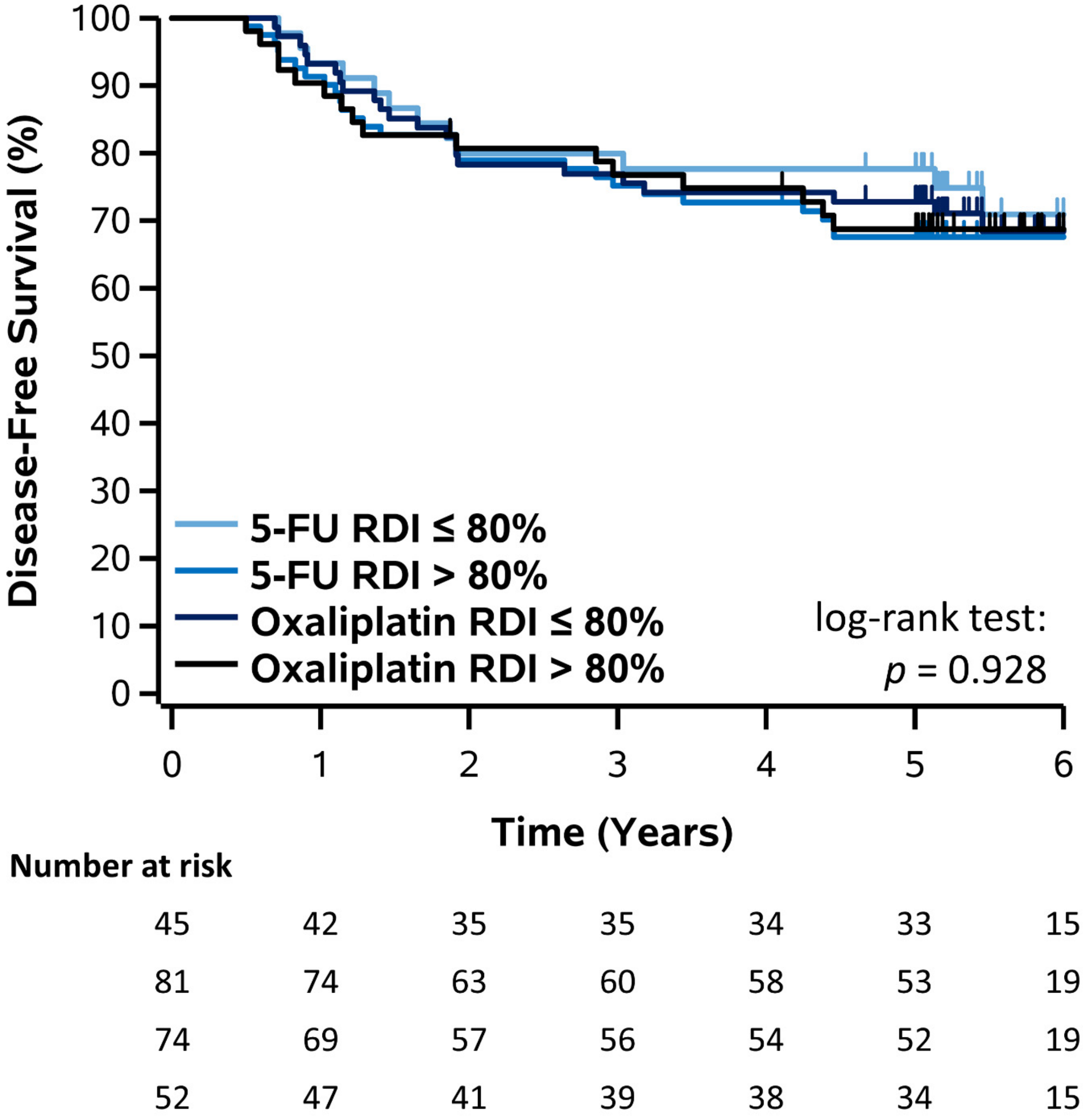

Figure S2. Kaplan–Meier plot of DFS stratified by RDI of 5-FU and oxaliplatin in patients receiving FOLFOX.

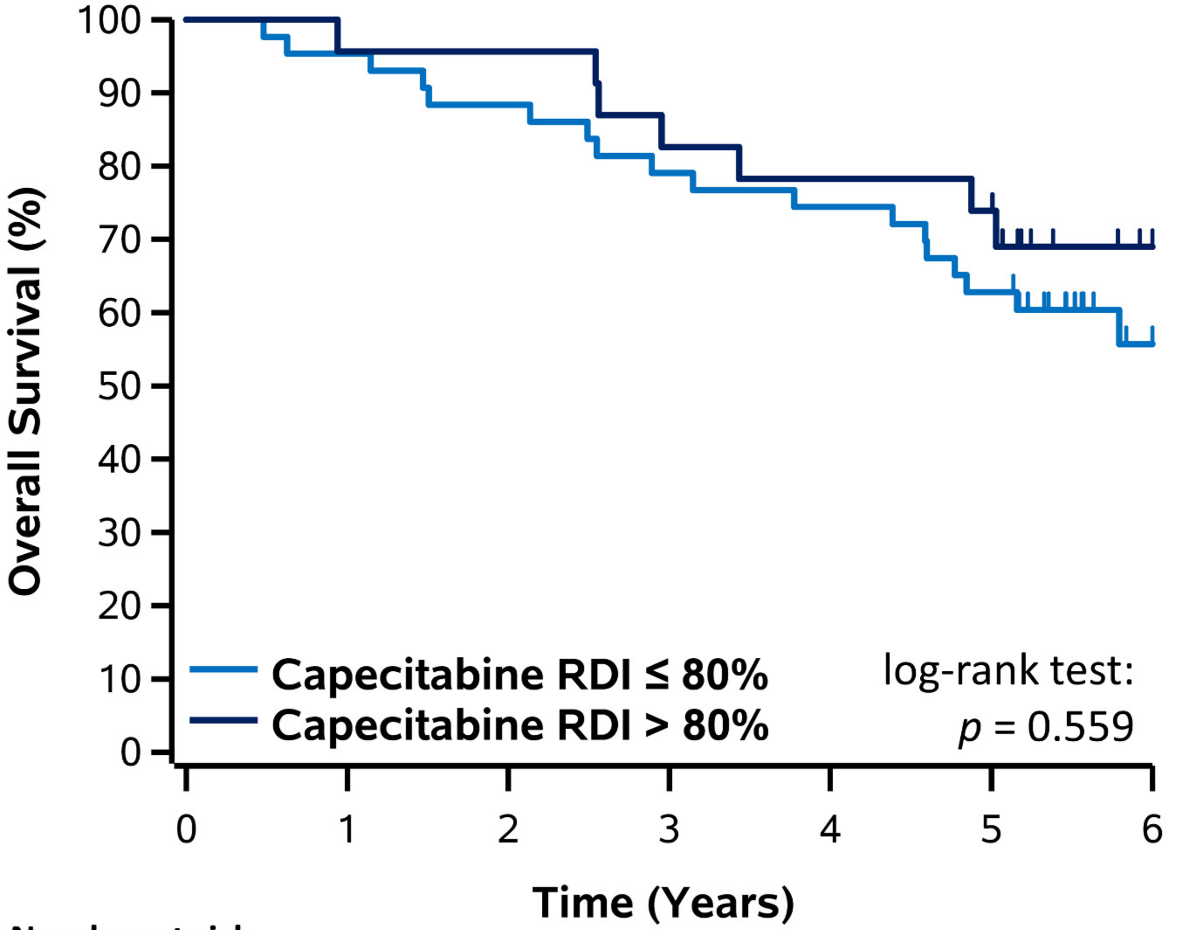

Number at risk

43	41	38	34	32	27	11
23	22	22	19	18	17	5

Figure S3. Kaplan–Meier plot of OS stratified by RDI of capecitabine in patients receiving capecitabine.

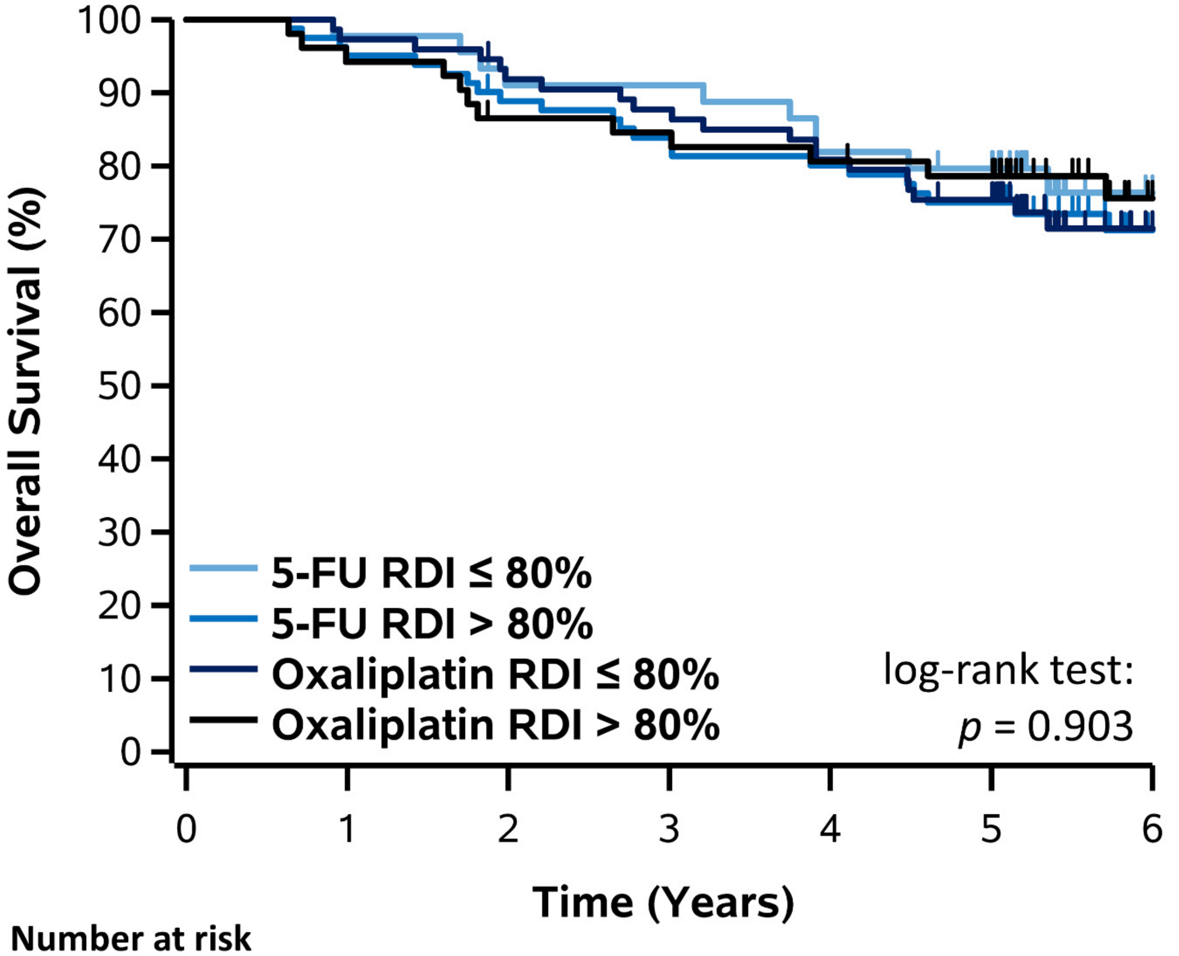

Number at risk

45	44	40	40	36	34	16
81	77	71	67	64	59	22
74	72	67	64	59	54	20
52	49	44	43	41	39	18

Figure S4. Kaplan–Meier plot of OS stratified by RDI of 5-FU and oxaliplatin in patients receiving FOLFOX.
